# The Mycobacterial Cell Envelope: A Relict From the Past or the Result of Recent Evolution?

**DOI:** 10.3389/fmicb.2018.02341

**Published:** 2018-10-09

**Authors:** Antony T. Vincent, Sammy Nyongesa, Isabelle Morneau, Michael B. Reed, Elitza I. Tocheva, Frederic J. Veyrier

**Affiliations:** ^1^INRS-Institut Armand-Frappier, Bacterial Symbionts Evolution, Laval, QC, Canada; ^2^McGill International TB Centre, Montreal, QC, Canada; ^3^Faculty of Dentistry, Université de Montréal, Montreal, QC, Canada; ^4^Department of Medicine, McGill University, Montreal, QC, Canada; ^5^Infectious Diseases and Immunity in Global Health Program, Research Institute of the McGill University Health Centre, Montreal, QC, Canada

**Keywords:** cell envelope, *Actinobacteria*, *Mycobacterium*, evolution, genomics

## Abstract

Mycobacteria are well known for their taxonomic diversity, their impact on global health, and for their atypical cell wall and envelope. In addition to a cytoplasmic membrane and a peptidoglycan layer, the cell envelope of members of the order *Corynebacteriales*, which include *Mycobacterium tuberculosis*, also have an arabinogalactan layer connecting the peptidoglycan to an outer membrane, the so-called “mycomembrane.” This unusual cell envelope composition of mycobacteria is of prime importance for several physiological processes such as protection from external stresses and for virulence. Although there have been recent breakthroughs in the elucidation of the composition and organization of this cell envelope, its evolutionary origin remains a mystery. In this perspectives article, the characteristics of the cell envelope of mycobacteria with respect to other actinobacteria will be dissected through a molecular evolution framework in order to provide a panoramic view of the evolutionary pathways that appear to be at the origin of this unique cell envelope. In combination with a robust molecular phylogeny, we have assembled a gene matrix based on the presence or absence of key determinants of cell envelope biogenesis in the *Actinobacteria* phylum. We present several evolutionary scenarios regarding the origin of the mycomembrane. In light of the data presented here, we also propose a novel alternative hypothesis whereby the stepwise acquisition of core enzymatic functions may have allowed the sequential remodeling of the external cell membrane during the evolution of *Actinobacteria* and has led to the unique mycomembrane of slow-growing mycobacteria as we know it today.

**GRAPHICAL ABSTRACT:**
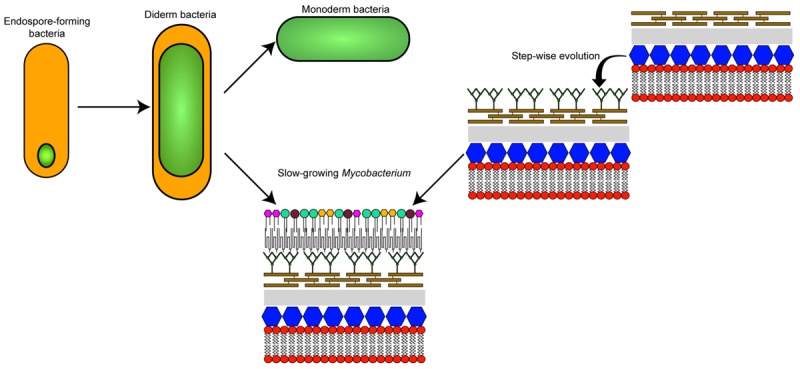
The different theories on the evolutionary origin of the mycobacterial cell envelope.

## Introduction

The *Actinobacteria* phylum of Gram-positive bacteria forms an extremely diverse group that includes several species that have evolved specific symbioses (commensal or parasitic) with a wide range of hosts including numerous mammals. For example, certain species from the genera *Mycobacterium* and *Nocardia* are pathogenic while others, belonging to the genus *Bifidobacterium*, are part of the normal gut microbial flora and are known to have a beneficial and important effect on human health (Barka et al., 2016; O’Callaghan and van Sinderen, 2016). Several *Actinobacteria* are also involved in the production of antibiotic compounds (e.g., *Streptomyces* sp.), amino acids (e.g., *Corynebacterium* sp.), biofuels, and other bioproducts (Becker and Wittmann, 2016; Lewin et al., 2016).

**FIGURE 1:**
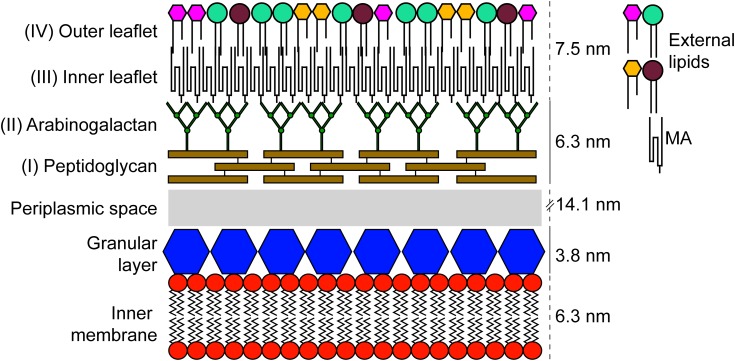
Schematic representation of the cell envelope found in members of the order *Corynebacteriales*. The components were scaled up using results obtained by CEMOVIS for *M. bovis* BCG (Zuber et al., 2008).

One of the most studied bacteria from the *Actinobacteria* phylum is *Mycobacterium tuberculosis*, the etiological agent of tuberculosis, a disease that causes significant morbidity and mortality. It is a leading cause of death worldwide making its control a top priority for the World Health Organization (Pai et al., 2016; Friedrich, 2017). Several bacteria from the *Corynebacteriales* order, that includes *M. tuberculosis*, are studied for having an atypical structural characteristic: the presence of a so-called “mycomembrane” that, in an organizational sense, is believed to resemble the outer membrane of typical Gram-negative bacteria (Zuber et al., 2008; Touchette and Seeliger, 2017; **Figure 1**). This mycomembrane is limited to members of the *Corynebacteriales* with some species-specific variation (Goodfellow and Jones, 2015; Mohammadipanah and Dehhaghi, 2017). Besides the components common to other Gram-positive bacteria, *Corynebacteriales* also have a layer of arabinogalactan attached to the peptidoglycan layer and to the inner leaflet of the mycolic-acid containing mycomembrane. This unusual structure, along with some phylogenetic ambiguity, have led certain authors to suggest that *M. tuberculosis* has more in common with Gram-negative bacteria than with their Gram-positive relatives (Fu and Fu-Liu, 2002). In the 1970s, two distinct cell envelope cleavage planes were recorded for freeze-etched mycobacteria (Barksdale and Kim, 1977). These findings contributed to the original proposal for a mycobacterial outer membrane. Further unequivocal evidence was provided by labeling with selective fluorescent probes (Christensen et al., 1999). In fact, the debate about the existence and composition of the mycomembrane was partially resolved only 10 years ago when it was visualized by cryo-electron microscopy of vitreous sections (CEMOVIS) (Hoffmann et al., 2008; Zuber et al., 2008). The reasons for the evolution of this membrane are still not totally clear, although we know that it is important for several aspects of the virulence and intrinsic antibiotic resistance of pathogenic species such as *M. tuberculosis* (Forrellad et al., 2013; Becker and Sander, 2016). In addition, as this membrane (along with the rest of the cell envelope) is at the frontline of environmental interactions, it is expected that differences in the constitution of the mycomembrane are associated with adaptation to specific environments or ecological niches (Veyrier et al., 2009).

In this era of large-scale DNA sequencing (Vincent et al., 2017), we can now investigate and compare the genomic sequences of bacteria at an unprecedented rate (Land et al., 2015) and infer the key steps that have led to the development and modification of the various subcomponents of bacterial cells. In recent years, numerous actinobacterial genomes have been sequenced including a broad representation of each genus. As expected, analysis of these data revealed a great heterogeneity in terms of the many biological functions associated with these bacteria (Ventura et al., 2007; Gomez-Escribano et al., 2016). Based on this large amount of sequence information, it is now possible to carry out an evolutionary analysis of the cell envelope in order to dissect the genetic events that have led to its development. Here, we describe the different layers of the cell envelope for the *Actinobacteria* phylum with a specific emphasis on species that harbor the mycomembrane. Although a number of mysteries still remain, our goal for this article is to provide new perspectives on the evolutionary path and benefits surrounding the unique membrane features of this important group of bacteria.

## Actinobacterial Cell Envelope Layers

### Granular Layer

By using CEMOVIS, it is possible to visualize a “granular layer” in between the plasma membrane and the peptidoglycan layer of *Mycobacterium bovis* BCG, *Mycobacterium smegmatis*, and *Corynebacterium glutamicum* (Zuber et al., 2008). A previous study made on other Gram-positive bacteria has shown that the granular layer is possibly linked to the plasma membrane and composed of penicillin-binding proteins, lipoproteins, and lipoteichoic acids (or teichuronic acid in some species; for review see Tul’skaya et al., 2011) (Zuber et al., 2006). Although, it seems this structure is common to several Gram-positive bacteria, the function and its precise composition are poorly characterized.

### Layer I: Peptidoglycan

The cell envelope of *Actinobacteria* is composed of a layer of peptidoglycan that provides essential functions such as rigidity and helps to maintain an optimal osmotic stability (Vollmer et al., 2008; Jankute et al., 2015). Although peptidoglycan is common amongst bacteria, there are many subtle differences in its composition that have been used in the past as a way to identify distinct species in the context of chemotaxonomy (Lechevalier and Lechevalier, 1970). The standard peptidoglycan consists of short peptides and glycan strands that are composed of *N*-acetylglucosamine (GlcNAc) and *N*-acetylmuramic acid (MurNAc) residues linked byβ-1→4 bonds. The third amino acid in the peptide stem is usually *meso*-diaminopimelic acid (*meso*-DAP) for Gram-negative and L-lysine for Gram-positive bacteria (Egan et al., 2017). In *Actinobacteria*, this amino acid is variable with numerous species (including the *Corynebacteriales* order) that harbor *meso*-DAP. This property is potentially due to the species-specific affinity of the ligase MurE (UDP-*N*-acetylmuramoylalanyl-D-glutamate-2,6-diaminopimelate ligase) for *meso*-DAP (Hammes et al., 1977; Basavannacharya et al., 2010). Furthermore, differences in these third amino-acids were used as a marker for *Actinobacteria* cell wall types II, III, and IV (Lechevalier and Lechevalier, 1970).

**FIGURE 2:**
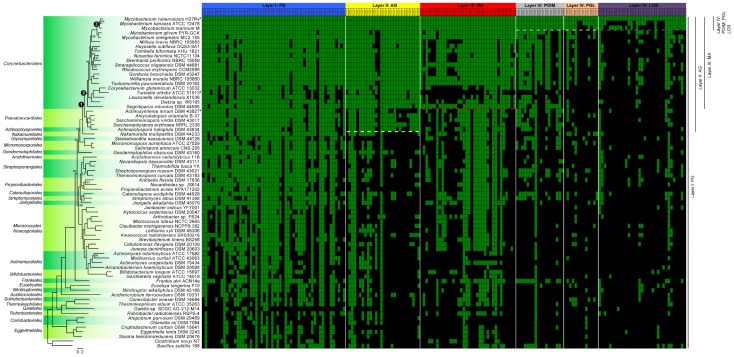
Distribution within the *Actinobacteria* phylum of genes involved in the synthesis of components of the cell envelope of *M. tuberculosis*. The 72 genome sequences (**Supplementary Table S1**), comprising at least one species per order, were annotated using PROKKA version 1.12 (Seemann, 2014). The phylogenetic tree was generated from 86 sequences inferred as orthologous by GET_HOMOLOGUES version 20180313 (Contreras-Moreira and Vinuesa, 2013). The sequences were aligned using MAFFT version 7.397 (Katoh and Standley, 2013) and concatenated and partitioned using AMAS (Borowiec, 2016). Finally, the best evolutionary model for each partition was found by IQ-TREE version 1.6.3 (Kalyaanamoorthy et al., 2017) and maximum-likelihood phylogenetic analysis was also performed using IQ-TREE (Nguyen et al., 2015) using 10,000 ultrafast bootstrap replicates (Hoang et al., 2018). Only bootstrap values under 100 are shown on the tree. Nodes that are discussed in the article are highlighted with a numbered black circle. The green and black squares of the matrix highlight genes that are present or absent, respectively. Individual genes (**Supplementary Table S2**) were considered to be present when they had a sequence similarity ≥50% relative to *M. tuberculosis* [an *e*-value cut-off of 1*e*^-10^ has also been applied in TBLASTN version 2.7.1 (Altschul et al., 1997)]. The raw data, containing the similarity values, is presented in the **Supplementary Table S2** for clarity. The “^∗^” represent micro-evolution of specific strains (described in the corresponding sections of the article). Dashed lines represent delineation, described in the literature, of the presence/absence of the specific layers also depicted at the right side of the figure. Of note, although the phylogenetic position of *Gaiella* sp. SCGC AG-212-M14 is expected for a member of the order *Gaiellales*, the size of the assembly (<1 Mb) coming from a single cell amplified by WGA-X, suggests that the sequence of this strain is likely incomplete.

In addition to having *meso*-DAP, the peptidoglycan of members of the *Corynebacteriales* order has another major distinction: a portion of MurNAc molecules is oxidized to become *N*-glycolylmuramic acid (MurNGlyc). This characteristic, in addition to playing an important role in the structure of peptidoglycan, has been suggested to increase resistance to lysozyme and β-lactam antibiotics (Raymond et al., 2005). The hydroxylase responsible for this modification is encoded by the gene *namH* (Rv3818; as per the reference *M. tuberculosis* H37Rv genome) (Raymond et al., 2005; Coulombe et al., 2009; Hansen et al., 2014). As expected, this gene is found in several members of the *Corynebacteriales* order (**Figure 2**). However, it appears to be absent from the genomes of genera such as *Hoyosella* and in those that are the most “basal” of the order: *Corynebacterium*, *Turicella*, *Lawsonella*, *Dietzia*, and *Segniliparus*. Interestingly, a few other genera in the *Actinobacteria* phylum, but not in the *Corynebacteriales*, also have a putative *namH* homolog (**Figure 2**). This is the case for *Stackebrandtia* (*Glycomycetales*), *Micromonospora* (*Micromonosporales*), and *Salinispora* (*Micromonosporales*) that form a discrete sub-lineage and have been described to harbor a cell wall with MurNGlyc (Parte et al., 2012). As an exception, a few scattered species (such as *Catenulispora acidiphila* and *Rubrobacter radiotolerans*) possess a homologous copy of NamH, although to the best of our knowledge there is no evidence for the presence of MurNGlyc in their peptidoglycan (Parte et al., 2012).

### Layer II: Arabinogalactan

In addition to its distinct composition, the peptidoglycan of *Corynebacteriales* is also the point of attachment of arabinogalactan, a highly branched heteropolysaccharide composed of galactose and arabinose in a furanoid form (**Figure 1**; McNeil et al., 1987). It is estimated that approximately 10% of the MurNAc residues of the peptidoglycan are covalently linked to arabinogalactan [_α_-_L_-rhamnopyranose-(1→3-α-D-GlycNAc-(1→P))] (McNeil et al., 1990). Although this linkage is unique, it resembles the one that connects cell wall teichoic acids to peptidoglycan in other Gram-positive species. This similarity led to the discovery of a phosphotransferase of the LytR-CpsA-Psr family, named Lcp1, which is encoded by the *Rv3267* gene and plays a leading role in linking arabinogalactan to peptidoglycan in *M. tuberculosis* (Baumgart et al., 2016; Grzegorzewicz et al., 2016; Harrison et al., 2016).

In terms of its distribution within the *Actinobacteria* phylum, arabinogalactan is produced exclusively by the members of the *Corynebacteriales* order and in some species from related orders such as *Pseudonocardiales* and *Actinopolysporales* (Goodfellow et al., 1984) [with the exception of *Actinosynnema mirum* (Hasegawa et al., 1978)]. These latter two orders possess a typical type IV cell wall (Lechevalier and Lechevalier, 1970), although they lack mycolic acids (Ludwig et al., 2015). The global distribution of genes implicated in the synthesis and transport of arabinogalactan is well correlated with the fact that these species produce arabinogalactan (see **Figure 2**). Nevertheless, a deeper investigation of these latter two orders will be required to exclude possible exceptions or misinterpretation.

### Layer III: Mycolic Acids

The inner leaflet of the external mycomembrane is homogeneous and is mainly composed of mycolic acids (MAs) – long-chain fatty acids that are exclusive to the order *Corynebacteriales* (Bansal-Mutalik and Nikaido, 2014; Marrakchi et al., 2014). On one hand, MAs form a barrier to hydrophilic molecules including several antibiotics (Liu et al., 1995), while on the other hand, they are also the target of some first- and second-line anti-TB drugs including isoniazid (Jackson et al., 2013; Marrakchi et al., 2014). In an evolutionary context, MAs play a prominent role in taxonomic differentiation within the *Corynebacteriales* order. In this sense, there is a great diversity in the number of carbons and functional groups comprising the MAs, thus making it possible to use them as a chemotaxonomic marker to differentiate between genera and also at the species level (Minnikin et al., 1984; Barry et al., 1998; Song et al., 2009; Marrakchi et al., 2014).

As reviewed elsewhere (Marrakchi et al., 2014), the raw materials of MAs, fatty acids, are produced by the combination of two fatty acid synthases (FAS-I and FAS-II). The first, FAS-I, is a eukaryotic-like protein narrowly distributed in bacteria (Cabruja et al., 2017) and encoded by the *fas* gene (Rv2524c). FAS-I produces acyl-CoAs with a bimodal distribution of C_16_-C_18_ and C_24_-C_26_. FAS-II, on the other hand, is far more common across bacterial species (Cabruja et al., 2017) and begins with the formation of β-ketoacyl-ACP by Claisen condensation of malonyl-ACP [produced from malonyl-CoA by MtFabD (Rv2243)], with acyl-CoA (the product of FAS-I), through the action of MtFabH (Rv0533). Other enzymes such as HadA (Rv0635), HadB (Rv0636), HadC (Rv0637), InhA (Rv1484), KasA (Rv2245), and KasB (Rv2246) are involved in the subsequent steps of elongation and maturation of fatty acids by FAS-II. The final MA structure is produced though several crucial steps that include, among others, activation (FadD32 and Rv3801c), condensation (Pks13 and Rv3800c) and reduction (CmrA and Rv2509).

Although the presence of MAs is well-conserved throughout the *Corynebacteriales*, there are some exceptions. For example, basal species of *Corynebacteriales* (such as *Corynebacterium*, *Dietzia, Lawsonella, and Turicella*) that form a monophyletic group, lack multiple genes confirming the well described diversity in length and composition of MA. It has already been reported that some species of *Corynebacterium* possess two FAS-I genes (such as *C*. *glutamicum*), while some do not have the genes encoding for the typical FAS-II machinery (Radmacher et al., 2005). Interestingly, *Corynebacterium* cannot elongate MAs (Radmacher et al., 2005; Burkovski, 2013), which is consistent with the lack of more than 50% of genes implicated in this pathway.

*Turicella otitidis* possesses almost none of the genes involved in MA biosynthesis (see “^∗^” in **Figure 2**). This species is clearly in the order *Corynebacteriales* based on its genome sequence (Baek et al., 2018). However, a recent study suggests that the genome of *T. otitidis* has lost the key genes involved in MA synthesis (Baek et al., 2018). This result corroborates the fact that this bacterium does not produce MAs which is exceptional for a bacterium of the order *Corynebacteriales* and this microevolution has made its positioning within this order rather difficult (Funke et al., 1994). Interestingly, this species still produces arabinogalactan (Renaud et al., 1996).

### Layer IV: External Lipids

While the composition of the inner leaflet of the mycomembrane is homogenous, the outer leaflet is highly heterogeneous and consists of lipids, lipoglycans, and proteins (Chiaradia et al., 2017). Due to its complexity and species-specific constitution, it remains poorly characterized with the exception of the pathogen *M. tuberculosis* and closely related slow-growing mycobacteria. In these bacteria, several lipids are associated with the external leaflet including – but not limited to – phthiocerol dimycocerosate (PDIM), phenolic glycolipid (PGL, phenolphthiocerol-based glycolipids that share a similar long-chain fatty acid backbone with PDIM), and lipooligosaccharides (LOS). These components seem to be important virulence factors (Reed et al., 2004; Forrellad et al., 2013) and have been shown to be involved in the infection of macrophages as well as in escape from the immune system (Cambier et al., 2014). The PDIM and PGL-associated genes (Rv2928 to Rv2963), are all co-located within the same cluster of the chromosome (Goude and Parish, 2008; **Supplementary Table S2**). LOS are common to several slow growing mycobacteria (including “*M. canettii*” from the *M. tuberculosis* complex) although they are not produced by *M. tuberculosis* due to the loss of Pks5.1 and truncation of PapA4. The remaining genes implicated in this pathway are present (**Figure 2**) which suggests micro-evolution in this species has led to the loss of LOS production (Boritsch et al., 2016; Brennan, 2016). This apparent evolutionary change correlates with a marked increase in whole cell hydrophobicity and enhanced aerosol transmission of current TB strains (Jankute et al., 2017).

The external layer of the mycomembrane also contains trehalose monomycolate – TMM, and trehalose dimycolate – TDM (also called “cord factor”) that also have crucial functions in the regulation of the host-symbiont relationship (Hunter et al., 2006). These molecules consist of glucose disaccharides (α-D-glucopyranosyl-α-D-glucopyranoside) esterified with MAs. TMM is generated in the cytoplasm, whereupon MmpL3 (Rv0206c) subsequently transports this molecule to the periplasm (Grzegorzewicz et al., 2016). Finally, the accepted model (for a review see Marrakchi et al., 2014) is that TMM in the periplasm serves as the MA donor for the mycolylation of arabinogalactan, and is also processed to give TDM. Both reactions involve the Ag85 enzyme complex (Belisle et al., 1997). Three genes (Rv3803c, Rv1886c, and Rv0129c) encode for the Ag85 complex and they are only present in the MA-positive species as seen in **Figure 2**. As TMM and TDM rely on the presence of MAs and Ag85, their distribution is therefore restricted to MA positive species. Indeed, they have been isolated from *Mycobacterium*, *Corynebacterium*, *Nocardia, and Rhodococcus* (Noll et al., 1956; Ioneda et al., 1970; Jean-Claude et al., 1976; Mompon et al., 1978; Lopes Silva et al., 1979; Pommier and Michel, 1979; Rapp et al., 1979; Batrakov et al., 1981). However, further characterization is necessary for other MA-positive species (such as *Segniliparus*).

## What Is the Evolutionary Origin of the Mycobacterial Cell Envelope?

The biological functions and molecular mechanisms surrounding the biosynthesis of the mycomembrane present in the *Corynebacteriales* are being uncovered slowly, but surely. However, the precise origin of this feature that is unique amongst members of the *Actinobacteria* (otherwise composed essentially of monoderm bacteria) continues to be a mystery. Two main theories can potentially explain the biogenesis of the mycomembrane: (1) that it arose via the remodeling of an already existing outer membrane, or (2) that it has a *de novo* origin (**Graphical Abstract**).

The first theory was proposed based on the observation that a double membrane is generated as a byproduct of endospore formation in bacteria (although endospores have not been found in *Actinobacteria*) (Tocheva et al., 2011; Tocheva et al., 2013). Assuming that the primordial cell was monoderm, a double membraned cell could have appeared via the retention of the second spore membrane, thus giving rise to a diderm common ancestor of all bacteria. This theory implies a bottleneck that selected for this ancestor but also that the current monoderm species appeared by independent loss of the outer membrane. In order to generate a mycomembrane, a significant re-engineering of the gene content (difficult to assess due to gene erosion over time) would have occurred in *Corynebacteriales* and could account for the atypical envelope characteristics when compared to other diderm species (Tocheva et al., 2016). This theory remains an open question in the field (Sutcliffe and Dover, 2016).

The second theory proposes that the double bacterial membrane is a homoplastic character (i.e., a similar trait from different evolutionary origins) that has evolved several times independently in a functionally convergent manner. With regard to the *Actinobacteria* phylum, the double membrane could have appeared “recently” by successive horizontal acquisition of genes allowing a step-by-step construction of the cell envelope that is present in *Mycobacterium*.

The distribution of the major genes implicated in the synthesis of the different layers of the mycomembrane is presented for the *Actinobacteria* in **Figure 2**. This figure needs to be interpreted carefully as the function of the different orthologous proteins has not been proven to be conserved across all the listed species. Nevertheless, one can observe a “phylogenetic gradient” in terms of the distribution of genes involved in the construction of the cell-envelope from inside to outside (**Figure 2**). In addition, one can observe a step-wise distribution in the evolution of the layers comprising the cell envelope. Both the gene content and the cell wall characteristics are well correlated. When investigating the genes involved in peptidoglycan synthesis throughout the genomes of the *Actinobacteria*, it can be observed that they are broadly distributed. On the other hand, arabinogalactan (and the genes implicated in this pathway) are restricted to bacteria that have diverged after the evolutionary node that we called “Node 1” (**Figure 2**). The MAs are even more narrowly distributed within bacteria that diverged after Node 2 (if we exclude the polyvalent FAS-II machinery). Finally, the genes associated with synthesis of the external leaflet are present in a sublineage-specific manner within the slow-growing mycobacteria that diverged after Node 3. This step-wise gradient of gene acquisition would support the theory in which the sequential gain-of-function has led to the successive evolution of the outer-membrane components in the ancestors of modern *M. tuberculosis*. The first step in this pathway appears to be the ability to attach and produce arabinogalactan that, in turn, supports the addition of the mycomembrane. Within this scenario, the final layer that corresponds to the external leaflet would have evolved most recently in a species-specific manner.

In this article, *M. tuberculosis* (and other slow growing mycobacteria) are used as our end point as they are the best characterized. However, we expect that with further characterization of the cell envelope of other *Corynebacteriales* and identification of the genes involved in their synthesis, we will obtain a similar general pattern. This will be particularly interesting for the highly heterogeneous outer leaflet. In fact, *Corynebacteriales* colonize multiple ecological niches from extreme and diverse habitats including soil and the human microbiome (Ramakrishnan et al., 2013; Goodfellow and Jones, 2015). This diversity implies a specific adaptation for individual species. This phenomenon would involve a distinctive selection of cell envelope constituents and, more specifically, for the external leaflet of the outer-membrane that is in immediate contact with the environment. For example, **Figure 2** shows that several genes involved in PDIM formation are present in the genomes of members of the *Corynebacteriales* order that are assumed to be PDIM-negative. This is especially true for *M. gilvum*, which is a rapid-growing mycobacterium (Wee et al., 2017). It is important to note that a number of genes involved in PDIM and PGL synthesis are polyketide synthases, which are versatile enzymes involved in the production of various natural products (Quadri, 2014). The question remains whether these genes serve in the production of molecules localized in the outer leaflet of “DIM-negative” bacteria that may have a common origin with PDIM as we know it in the pathogenic slow-growing mycobacteria.

## Conclusion

Regardless of whether the double membrane of *Corynebacteriales* represents a relict sharing a common origin with the double membrane present in typical Gram-negative species, or is the result of a more recent adaptation, it will undoubtedly be interesting and informative to attempt to experimentally reconstruct the double membrane of mycobacteria starting from an *Actinobacteria* species possessing a single membrane (such as *Turicella* sp.). This daring experiment will teach us more about the mechanism of biogenesis of this double membrane and, perhaps, even inform on its molecular evolution and the natural selection of such an adaptation. In doing so, we could mimic the putative evolutionary process that has led to the *Mycobacterium* membrane as well as identify the functional differences linked to possible species-specific adaptation of the mycomembrane. This work may also highlight important enzyme functions that can be specifically targeted in vaccination or chemotherapeutic approaches aimed at killing pathogenic species such as *M. tuberculosis*.

## Author Contributions

ATV and FJV conceived and designed the experiments. ATV, FJV, MR, ET, SN, and IM contributed to the writing and editing of the manuscript. All authors read and approved the final manuscript.

## Conflict of Interest Statement

The authors declare that the research was conducted in the absence of any commercial or financial relationships that could be construed as a potential conflict of interest.
