# Supplementary material for: The Mycobacterial Cell Envelope: A Relict From the Past or the Result of Recent Evolution?
Source: Front Microbiol. 2018 Oct 9;9:2341. doi: 10.3389/fmicb.2018.02341 (PMC6194230; doi:10.3389/fmicb.2018.02341)
Supplement: Supplementary file 1 [file Table_1.DOCX]

Supplementary Material

The mycobacterial cell envelope: a relict from the past or the result of recent evolution?

Antony T. Vincent^1,5^, Sammy Nyongesa^1^, Isabelle Morneau^2^, Michael B. Reed^3,4,5^, Elitza I. Tocheva^2^, Frederic J. Veyrier^1,5*^

^1^INRS-Institut Armand-Frappier, Bacterial Symbionts Evolution, Laval, Quebec, Canada.

^2^Université de Montréal, Faculty of Dentistry, C.P. 6128, succursale Centre-ville, Montreal, Quebec, Canada.

^3^Department of Medicine, McGill University, Montreal, Quebec, Canada.

^4^Infectious Diseases & Immunity in Global Health Program, Research Institute of the McGill University Health Centre, Montreal, Quebec, Canada

^5^McGill International TB Centre, Montreal, Quebec, Canada

*** Correspondence:** Frédéric J. Veyrier: frederic.veyrier@iaf.inrs.ca

# Supplementary Table

**Table S1**. Genomic sequences used in this study

| **Order** | **Genus** | **Species** | **Strain** | **Assembly** | **Size (Mb)** | **GC %** | **Scaffolds** |
| --- | --- | --- | --- | --- | --- | --- | --- |
| *Corynebacteriales* | *Mycobacterium* | *tuberculosis* | H37Rv | GCA_000195955.2 | 4,41 | 65,6 | 1 |
| *Corynebacteriales* | *Mycobacterium* | *kansasii* | ATCC 12478 | GCA_000157895.2 | 6,58 | 66,19 | 2 |
| *Corynebacteriales* | *Mycobacterium* | *marinum* | M | GCA_000018345.1 | 6.66 | 65,71 | 2 |
| *Corynebacteriales* | *Mycobacterium* | *gilvum* | PYR-GCK | GCA_000016365.1 | 5,98 | 67,73 | 4 |
| *Corynebacteriales* | *Mycobacterium* | *smegmatis* | MC2 155 | GCA_000015005.1 | 6,99 | 67,4 | 1 |
| *Corynebacteriales* | *Millisia* | *brevis* | NBRC 105863 | GCA_001552615.1 | 5,65 | 69,2 | 53 |
| *Corynebacteriales* | *Hoyosella* | *subflava* | DQS3-9A1 | GCA_000214175.1 | 4,86 | 62,2 | 3 |
| *Corynebacteriales* | *Tomitella* | *biformata* | AHU 1821 | GCA_000524475.1 | 4,71 | 68,1 | 113 |
| *Corynebacteriales* | *Nocardia* | *farcinica* | NCTC11134 | GCA_001182745.2 | 6,46 | 70,61 | 5 |
| *Corynebacteriales* | *Skermania* | *piniformis* | NBRC 15059 | GCA_001552855.1 | 4,21 | 68,6 | 44 |
| *Corynebacteriales* | *Smaragdicoccus* | *niigatensis* | DSM 44881 | GCA_000380645.1 | 5,32 | 64,3 | 19 |
| *Corynebacteriales* | *Rhodococcus* | *erythropolis* | CCM2595 | GCA_000454045.1 | 6,37 | 62,50 | 2 |
| *Corynebacteriales* | *Gordonia* | *bronchialis* | DSM 43247 | GCA_000024785.1 | 5,29 | 67,07 | 2 |
| *Corynebacteriales* | *Williamsia* | *muralis* | NBRC 105860 | GCA_001598915.1 | 5,54 | 64,7 | 22 |
| *Corynebacteriales* | *Tsukamurella* | *paurometabola* | DSM 20162 | GCA_000092225.1 | 4,48 | 68,39 | 2 |
| *Corynebacteriales* | *Corynebacterium* | *glutamicum* | ATCC 13032 | GCA_000011325.1 | 3,31 | 53,8 | 1 |
| *Corynebacteriales* | *Turicella* | *otitidis* | ATCC 51513 | GCA_000296405.1 | 2,12 | 71 | 6 |
| *Corynebacteriales* | *Lawsonella* | *clevelandensis* | X1036 | GCA_001293125.1 | 1,86 | 58,6 | 1 |
| *Corynebacteriales* | *Dietzia* | sp. | W5195 | GCA_002999155.1 | 3,68 | 70,73 | 3 |
| *Corynebacteriales* | *Segniliparus* | *rotundus* | DSM 44985 | GCA_000092825.1 | 3,16 | 66,8 | 1 |
| *Pseudonocardiales* | *Actinosynnema* | *mirum* | DSM 43827 | GCA_000023245.1 | 8,25 | 73,7 | 1 |
| *Pseudonocardiales* | *Amycolatopsis* | *orientalis* | B-37 | GCA_000943515.2 | 9,49 | 68,8 | 1 |
| *Pseudonocardiales* | *Saccharomonospora* | *viridis* | DSM 43017 | GCA_000023865.1 | 4,31 | 67,3 | 1 |
| *Pseudonocardiales* | *Saccharopolyspora* | *erythraea* | NRRL2338 | GCA_000062885.1 | 8,21 | 71,1 | 1 |
| *Actinopolysporales* | *Actinopolyspora* | *halophila* | DSM 43834 | GCA_000371785.1 | 5,35 | 68 | 2 |
| *Nakamurellales* | *Nakamurella* | *multipartita* | DSM 44233 | GCA_000024365.1 | 6,06 | 70,9 | 1 |
| *Glycomycetales* | *Stackebrandtia* | *nassauensis* | DSM 44728 | GCA_000024545.1 | 6,84 | 68,1 | 1 |
| *Micromonosporales* | *Micromonospora* | *aurantiaca* | ATCC 27029 | GCA_000145235.1 | 7,03 | 72,8 | 1 |
| *Micromonosporales* | *Salinispora* | *arenicola* | CNS-205 | GCA_000018265.1 | 5,79 | 69,5 | 1 |
| *Geodermatophilales* | *Geodermatophilus* | *obscurus* | DSM 43160 | GCA_000025345.1 | 5,32 | 74 | 1 |
| *Acidothermales* | *Acidothermus* | *cellulolyticus* | 11B | GCA_000015025.1 | 2,44 | 66,9 | 1 |
| *Streptosporangiales* | *Nocardiopsis* | *dassonvillei* | DSM 43111 | GCA_000092985.1 | 6,54 | 72,71 | 2 |
| *Streptosporangiales* | *Thermobifida* | *fusca* | YX | GCA_000012405.1 | 3,64 | 67,5 | 1 |
| *Streptosporangiales* | *Streptosporangium* | *roseum* | DSM 43021 | GCA_000024865.1 | 10,37 | 70,9 | 2 |
| *Streptosporangiales* | *Thermomonospora* | *curvata* | DSM 43183 | GCA_000024385.1 | 5,64 | 71,6 | 1 |
| *Propionibacteriales* | *Kribbella* | *flavida* | DSM 17836 | GCA_000024345.1 | 7,58 | 70,6 | 1 |
| *Propionibacteriales* | *Nocardioides* | sp. | JS614 | GCA_000015265.1 | 5,29 | 71,48 | 2 |
| *Propionibacteriales* | *Propionibacterium* | *acnes* | KPA171202 | GCA_000008345.1 | 2,56 | 60 | 1 |
| *Catenulisporales* | *Catenulispora* | *acidiphila* | DSM 44928 | GCA_000024025.1 | 10,47 | 69,8 | 1 |
| *Streptomycetales* | *Streptomyces* | *albus* | DSM 41398 | GCA_000827005.1 | 8,38 | 72,6 | 1 |
| *Jiangellales* | *Jiangella* | *alkaliphila* | DSM 45079 | GCA_900105925.1 | 7,72 | 72,1 | 1 |
| *Micrococcales* | *Janibacter* | *indicus* | YFY001 | GCA_001889125.1 | 3,4 | 71,2 | 1 |
| *Micrococcales* | *Kytococcus* | *sedentarius* | DSM 20547 | GCA_000023925.1 | 2,79 | 71,6 | 1 |
| *Micrococcales* | *Arthrobacter* | sp. | FB24 | GCA_000196235.1 | 5,07 | 65,42 | 4 |
| *Micrococcales* | *Micrococcus* | *luteus* | NCTC 2665 | GCA_000023205.1 | 2,5 | 73 | 1 |
| *Micrococcales* | *Clavibacter* | *michiganensis* | NCPPB 382 | GCA_000063485.1 | 3,4 | 72,53 | 3 |
| *Micrococcales* | *Leifsonia* | *xyli* | DSM 46306 | GCA_000470775.1 | 2,69 | 68,3 | 1 |
| *Kineosporiales* | *Kineococcus* | *radiotolerans* | SRS30216 | GCA_000017305.1 | 4,96 | 74,21 | 3 |
| *Micrococcales* | *Brevibacterium* | *linens* | BS258 | GCA_001606005.1 | 3,86 | 64,2 | 1 |
| *Micrococcales* | *Cellulomonas* | *flavigena* | DSM 20109 | GCA_000092865.1 | 4,12 | 74,3 | 1 |
| *Micrococcales* | *Jonesia* | *denitrificans* | DSM 20603 | GCA_000024065.1 | 2,75 | 58,4 | 1 |
| *Actinomycetales* | *Actinomyces* | *odontolyticus* | ATCC 17982 | GCA_000154225.1 | 2,39 | 65,4 | 2 |
| *Actinomycetales* | *Mobiluncus* | *curtisii* | ATCC 43063 | GCA_000196535.1 | 2,15 | 55,4 | 1 |
| *Actinomycetales* | *Actinomyces* | *urogenitalis* | DSM 15434 | GCA_000159035.1 | 2,7 | 68 | 67 |
| *Actinomycetales* | *Arcanobacterium* | *haemolyticum* | DSM 20595 | GCA_000092365.1 | 1,99 | 53,1 | 1 |
| *Bifidobacteriales* | *Bifidobacterium* | *longum* | ATCC 15697 | GCA_000020425.1 | 2,83 | 59,9 | 1 |
| *Bifidobacteriales* | *Gardnerella* | *vaginalis* | ATCC 14019 | GCA_000159155.2 | 1,67 | 41,4 | 1 |
| *Frankiales* | *Frankia* | *alni* | ACN14A | GCA_000058485.1 | 7,5 | 72,8 | 1 |
| *Euzebyales* | *Euzebya* | *tangerina* | F10 | GCA_003074135.1 | 4.78 | 54,58 | 12 |
| *Nitriliruptorales* | *Nitriliruptor* | *alkaliphilus* | DSM 45188 | GCA_000969705.1 | 5,56 | 72,1 | 1 |
| *Acidimicrobiales* | *Acidimicrobium* | *ferrooxidans* | DSM 10331 | GCA_000023265.1 | 2,16 | 68,3 | 1 |
| *Solirubrobacterales* | *Conexibacter* | *woesei* | DSM 14684 | GCA_000025265.1 | 6,36 | 72,7 | 1 |
| *Thermoleophilales* | *Thermoleophilum* | *album* | ATCC 35263 | GCA_900108055.1 | 2,21 | 69,1 | 5 |
| *Gaiellales* | *Gaiella* | sp. | SCGC AG-212-M14 | GCA_001644475.1 | 1 | 66,4 | 82 |
| *Rubrobacterales* | *Rubrobacter* | *radiotolerans* | RSPS-4 | GCA_000661895.1 | 3,27 | 66,75 | 4 |
| *Coriobacteriales* | *Atopobium* | *parvulum* | DSM 20469 | GCA_000024225.1 | 1,54 | 45,7 | 1 |
| *Coriobacteriales* | *Olsenella* | *uli* | DSM 7084 | GCA_000143845.1 | 2,05 | 64,7 | 1 |
| *Eggerthellales* | *Cryptobacterium* | *curtum* | DSM 15641 | GCA_000023845.1 | 1,62 | 50,9 | 1 |
| *Eggerthellales* | *Eggerthella* | *lenta* | DSM 2243 | GCA_000024265.1 | 3,63 | 64,2 | 1 |
| *Eggerthellales* | *Slackia* | *heliotrinireducens* | DSM 20476 | GCA_000023885.1 | 3,17 | 60,2 | 1 |
| *Clostridiales* | *Clostridium* | *novyi* | NT | GCA_000014125.1 | 2,55 | 28,9 | 1 |
| *Bacillales* | *Bacillus* | *subtilis* | 168 | GCA_000009045.1 | 4,22 | 43,5 | 1 |
